# Editorial: Bridging the Theories of Affordances and Limb Apraxia

**DOI:** 10.3389/fnhum.2017.00148

**Published:** 2017-03-29

**Authors:** Antonello Pellicano, Anna M. Borghi, Ferdinand Binkofski

**Affiliations:** ^1^Division for Clinical and Cognitive Sciences, Department of Neurology Medical Faculty, RWTH Aachen UniversityAachen, Germany; ^2^Department of Dynamic and Clinical Psychology, Sapienza University of RomeRome, Italy; ^3^Institute of Cognitive Sciences and Technologies, Italian National Research CouncilRome, Italy

**Keywords:** affordances, apraxias, stable/variable affordances, attention, language and affordances, handle-to-hand correspondence effect

Affordances are meaningful relations between the features of observed objects and the observer's action systems with its proper abilities. The notion of affordance integrates perceptual, cognitive and motor functions, so that perceiving an object, conducting cognitive operations on it, and executing motor actions with it cannot be considered as independent functions. Limb apraxia is a higher-order motor disorder that refers to disturbance of one or more of three domains: imitation of meaningless gestures, pantomime of meaningful gestures, and disturbance of interaction with objects. The first aim of the Research Topic was to put together theoretical and research contributions on affordance mechanisms to highlight their role in explaining apraxia deficits. The second aim was to clarify how studies on apraxia have implications for theories of affordances. Here we provide a summary of the contributions to the Research Topic. We will first discuss three issues related to the mechanisms underlying affordances and their implications for apraxia, then we will describe the studies directly focusing on apraxia.

## Broken handles and attention

Two studies investigated the role of attention in affordance perception for objects with broken handles. Ambrosecchia et al. investigated the *handle-to-hand correspondence effect* (CE) to support the affordance activation account, or the location coding account (attention-based Simon effect, see Pellicano et al., in press). A discrimination task was performed on graspable objects with intact and broken handles, preceded by a spatial Stimulus-Response Compatibility task with incompatible S-R mapping. The CE was eliminated with broken-handle objects, whereas it stayed significant with intact-handle objects. Thus, CE seems to depend on both affordance and attention mechanisms. Wulff and Humphreys also presented single objects and object-pairs (e.g., teapot + cup) with broken handles to patients with left visual extinction. In object-pairs the broken handle reduced the degree to which it captured attention, especially when the tool-object fell within the ipsilesional side. Thus, to facilitate affordance perception, patients should be trained on the contralesional side with action-pairs. Overall, both studies showed affordance effects that cannot be reduced to simple attentional effects.

## Stable and variable affordances

The second conceptual node addressed in the Topic revolves around the notion of stable/variable affordance, and its eventual implications for apraxia. Borghi and Riggio proposed this distinction, Mizelle and Wheaton defended it; Osiurak argued instead that apraxia is not a matter of affordances. Indeed, on the base of three assumptions, Osiurak claimed that his *mechanical knowledge hypothesis* represents an alternative to the manipulation knowledge/stable affordances hypothesis (Binkofski and Buxbaum, 2013). First: The conception of tool use is based on allocentric knowledge of abstract mechanical principles. Second: The semantic knowledge of objects and tools is another form of allocentric knowledge, linking together different tools and objects when used in the same context or for the same target. Third: Affordances only translate the allocentric representation of the tool action into precise egocentric motor programs. Osiurak concluded that tool use apraxia is not a matter of affordances, but also that there is no distinction between variable and stable affordances: affordances are necessarily stable, because they must fit to human, biomechanical capacities, but are also temporary because they are perceived only as part of a specific goal. Mizelle and Wheaton commented on their model for Modular Selection for Action Goals (MSAG) in light of Osiurak's and Pellicano et al.'s (2011) articles. They contended that their MSAG model provides a preliminary framework to relate conceptual and motor “faults” to each other, which would reflect conceptual and ideomotor apraxias.

Borghi and Riggio presented the distinction between stable and variable affordances (Borghi and Riggio, 2009; Sakreida et al., 2016), and responded to the objections raised by Osiurak: even if they are not dichotomous, stable affordances (represented in the ventro-dorsal stream) emerge from characteristics less variable across contexts, as objects' size, whereas variable affordances (dorso-dorsal stream) from characteristics that change across contexts, as objects orientation. They reported that, during offline linguistic tasks, stable rather than variable affordances are recruited (Borghi, 2012): in line with the theory of reuse (Gallese, 2008) language recruits and modifies pre-existent mechanisms of the motor system. The authors also discussed whether automatic activation of affordances is challenged by task and context modulations: Automaticity and contextual dependency/flexibility are not necessarily in conflict, since the context can operate as a late filter. Importantly, the stable/variable distinction can address the automaticity issue: in offline tasks stable affordances are automatically activated, but also modulated by the task/context; in online tasks variable affordances are first activated. Overall, the authors of the contributions of this section debated to what extent the distinction between stable and variable affordances has implications for apraxia.

## Language and affordances

Marino et al. investigated whether pictures and words of manipulable objects recruit the motor system in a similar way. They found slower responses with manipulable compared to non-manipulable objects independently from the responding hand. This cost is likely due to two concurrent tasks (i.e., stimulus processing and response production). The authors speculated that similar performance with nouns and photos can be either due to stable affordances being only coded, or to the fact that natural objects rather than tools were used. In his commentary, Makris contended that 150 ms after stimulus presentation is too early for an affordance effect to emerge. Makris argued that the effect could be attentional, and suggested an affordance competition interpretation (Cisek, 2007): graspable objects immediately catch exogenous attention, which are then redirected to non-graspable objects 150 ms after stimulus onset, leading to a rebalance of affordance-driven motor plans. Buccino and Marino recognize that the attentional hypothesis cannot be completely ruled-out; however it is unclear why attention would be captured only by graspable objects, since also non-graspable ones were presented abruptly. Bub et al. examined the influence of holding planned hand-actions in working memory for the time taken to identify handled objects. Their result suggested that the representation of the appropriate grasping action for one object is based on its canonical orientation, rather than on its contingently depicted orientation.

From their side, Taylor et al. found selective deficits in understanding motor action verbs in patients with lesions involving posterior, parietal, and lateral occipitotemporal cortex. In contrast, deficits in understanding motionless action verbs were found in patients with more anterior lesions sparing posterior parietal and lateral occipitotemporal cortex. They speculated that semantic representations for motion and motionless actions are behaviorally and neuro-anatomically dissociable. The findings presented in this section provide a hint toward the role of perceptual and motor regions in processing modality-specific semantic knowledge.

## Affordances and implications for apraxia

Michałowski and Króliczak criticized the fact that the understanding of tool representations is provided by investigations of right-handed individuals and their typical organization of cognitive and manual skills. They claimed that tool-related processing in left-handers with greater incidence of right-sided or bilateral (atypical) lateralization of functions is not just mirror reversed. Therefore, caution is required in neurorehabilitation directed at left-handed patients. Rounis and Humphreys based their mini review on the *affordance competition hypothesis* (Cisek, 2007). According to them, some aspects of apraxia may reflect an abnormal sensitivity to competition when multiple affordances are present and/or a poor ability to exert cognitive control over this competition when it occurs. This framework would help overcoming the distinction between ideomotor and ideational apraxia, and account for mixed symptoms from the two disorders.

Randerath and Frey scrutinized the role of affordance perception on feedback learning. Participants judged whether their hand would fit into a given aperture, and whether objects were reachable. Performance resulted worst for openings or distances close to the individual's physical limits. Feedback improved performance in both tasks suggesting a rapidly trainable affordance perception. Furthermore, feedback experience could transfer between hands.

Evans et al., tested the assumption that in Apraxia, stored object knowledge from the ventral stream is less readily available to incorporate into the action plan; leading to an over-reliance on visual affordances in object-directed motor behavior. Left-hemisphere stroke-patients, apraxia-patients, and healthy controls grasped cylindrical objects of varying weight distribution. Object weight was indicated by either a memory-associated or a visual-spatial cue. Apraxia-patients suggested impaired integration of visible and known object properties attributed to the ventro-dorsal stream. In learning to grasp the weighted object, they applied neither pure knowledge-based information (memory-associated condition) nor higher-level information (visual-spatial cue condition).

Canzano et al. review focused on how objects use helps to better understand apraxia. They considered transitive vs. intransitive action dissociation, and the less frequent *constructive* and *magnetic apraxia*. They also considered pantomime and objects imitation within a view to dissociating the various components involved in upper limb apraxia. They concluded that object knowledge and sensory-motor representations are further supported by spatial and body representations, executive functions, and monitoring systems.

In summary, the recent revival of the idea of affordances has led to further refinement of the concept, and opened new avenues to the understanding of the interaction with objects and tools. This development kindled the research on brain representations of affordances. This special Topic provides emerging evidence that affordances code flexibly the dynamic interaction with objects. It seems that Apraxia-patients are capable only to utilize very basic affordances. We can thus speculate that observable apraxic deficits derive from the inability to utilize the flexible features of affordances.

## Author contributions

AP, AB, and FB gave a substantial contributions to the conception and the design of the work, drafted the work, revised the manuscript critically for important intellectual contents, agreed to be accountable for all aspects of the work in ensuring that questions related to the accuracy or integrity of any part of the work are appropriately investigated and resolved, gave a final approval of the version to be published. AP edited the final version of the manuscript.

## Funding

This work was supported by the START-Programme der Medizinischen Fakultät der RWTH-Aachen (START-Projekt 691240, 144/12).

### Conflict of interest statement

The authors declare that the research was conducted in the absence of any commercial or financial relationships that could be construed as a potential conflict of interest.
